# Supplementary material for: IFN-γ-dependent NK cell activation is essential to metastasis suppression by engineered Salmonella
Source: Nat Commun. 2021 May 5;12:2537. doi: 10.1038/s41467-021-22755-3 (PMC8099885; doi:10.1038/s41467-021-22755-3)
Supplement: Supplementary file 1 — Supplementary Information [file 41467_2021_22755_MOESM1_ESM.pdf]

IFN-γ-dependent NK cell activation is essential to metastasis suppression by engineered *Salmonella*

Supplementary Information

Qiubin Lin[1,2,7], Li Rong[1,7], Xian Jia[3], Renhao Li[1,4], Bin Yu[1], Jingchu Hu[5], Xiao Luo[5], Badea SR[1], Chen Xu[1], Guofeng Fu[3], Kejiong Lai[3], Ming-chun Lee[1], Baozhong Zhang[5], Huarui Gong[1], Nan Zhou[5], Xiao Lei Chen[3,6], Shu-hai Lin[3,*], Guo Fu[3,6,*], Jian-Dong Huang[1,2,5,*]

[1]School of Biomedical Sciences, the University of Hong Kong, Pokfulam, Hong Kong SAR, China.
[2] HKU-Zhejiang Institute of Research and Innovation (HKU-ZIRI), Hangzhou, PR China.
[3]State Key Laboratory of Cellular Stress Biology, Innovation Center for Cell Signaling Network, School of Medicine, Xiamen University, Xiamen, China.
[4]Department of Medicine, Li Ka Shing Faculty of Medicine, The University of Hong Kong, Pokfulam, Hong Kong SAR, China.
[5]Institute of Synthetic Biology, Shenzhen Institutes of Advanced Technology, Chinese Academy of Sciences, Shenzhen 518055, PR China.
[6]Cancer Research Center of Xiamen University, Xiamen, China.
[7]These authors equally contribute to this study.

*: Correspondence should be addressed to J.D.H. (E-mail: jdhuang@hku.hk), S.H.L. (shuhai@xmu.edu.cn); and G.F. (guofu@xmu.edu.cn)

Supplementary figures and legends

Supplementary Fig. 1

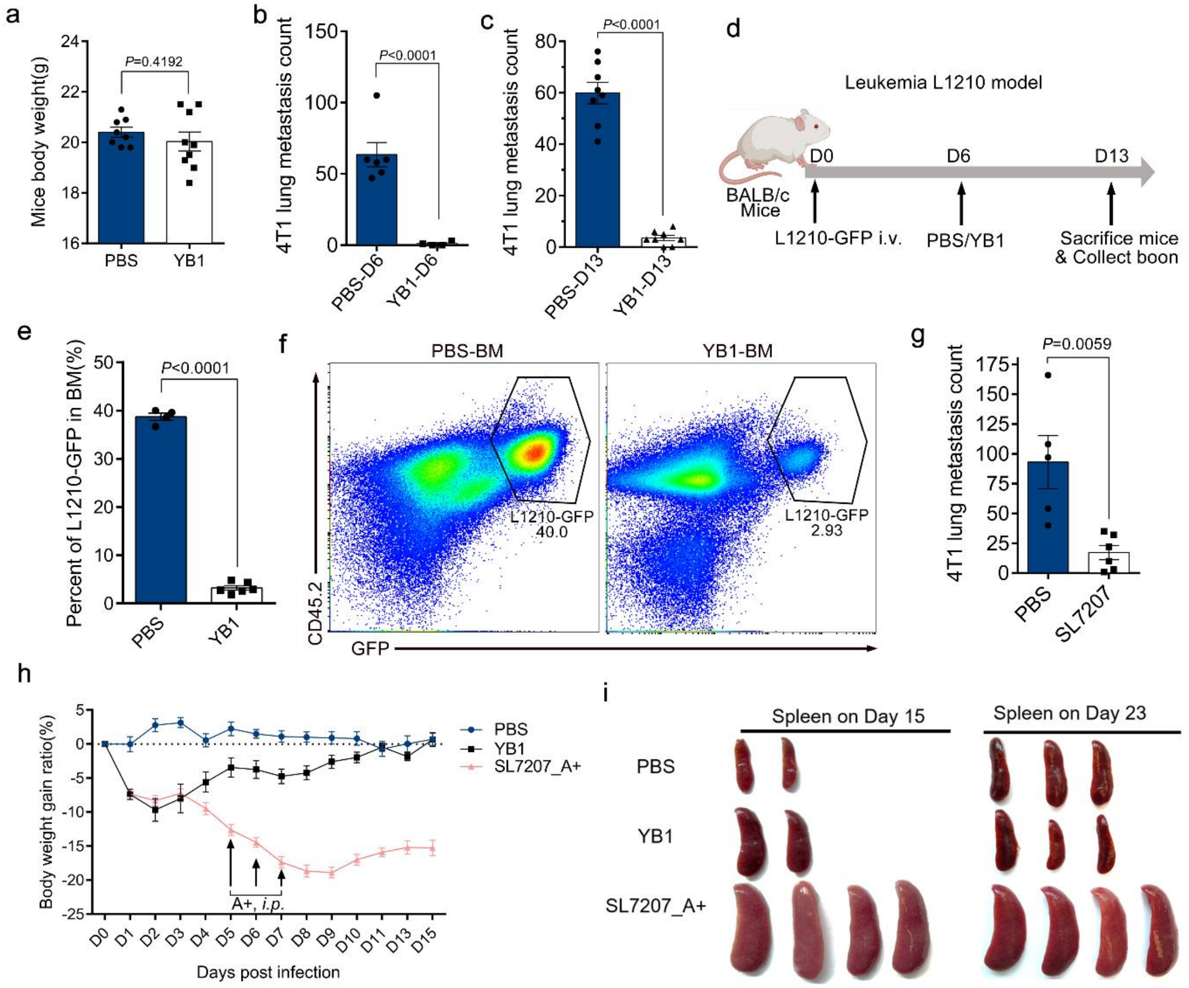

Supplementary Fig. 1 *Salmonella* YB1 treatment inhibits cancer metastasis and prolongs the survival of mice. a Mice body weight was measured before surgery to remove 4T1 primary tumors (n=8 PBS, n=9 YB1). **b** Quantification of lung metastases in the 4T1-BALB/c experimental metastasis model. 4T1 cells were i.v. injected on day 6 (n=6 mice) post *Salmonella* YB1 treatment. **c** Quantification of lung metastases in the 4T1-BALB/c experimental metastasis model. 4T1 cells were i.v. injected on day 13 (n=8 per group) post *Salmonella* YB1 treatment. **d** BALB/c mice were i.v. injected with 5×10^6 L1210-GFP cells on day 0 and treated with either *Salmonella* or PBS on day 6. All mice were sacrificed on day 13 to quantify the L1210-GFP cells in the bone marrow. **e** Quantification of the percentage of L1210-GFP cells to total immune cells in the bone marrow (n=4 in PBS group, n=6 in YB1 group). **f** Representative flow cytometry dot plots illustrating the percentage of L1210-GFP in the bone marrow of mice treated with PBS or YB1. **g** Quantification of lung metastases in the 4T1-BALB/c experimental metastasis model post *Salmonella* SL7207 treatment. Ampicillin was given to mice from day 3 to kill *Salmonella* (n=5 in PBS group, n=6 in SL7207 group).**h-i** 18 BALB/c mice were divided into three groups

and treated with PBS (n=5), 8x10^6 CFU of YB1 (n=5), or 8x10^6 CFU of SL7207(n=8), respectively. 100ul 100mg/ml ampicillin was given to each SL7207 treated mouse (SL7207_A+) on days 5, 6, and 7 by i.p. injection to control *salmonella* infection. Daily body weight changes were monitored before day 15 (**h**). Mice were killed at two timepoints to harvest spleen (**i**). *P*-values were derived using two-tailed unpaired t-tests. All data are shown as the mean values +/- s.e.m. Displayed is one representative experiment of 2 independent experiments. Source data are provided as a Source Data file.

Supplementary Fig. 2

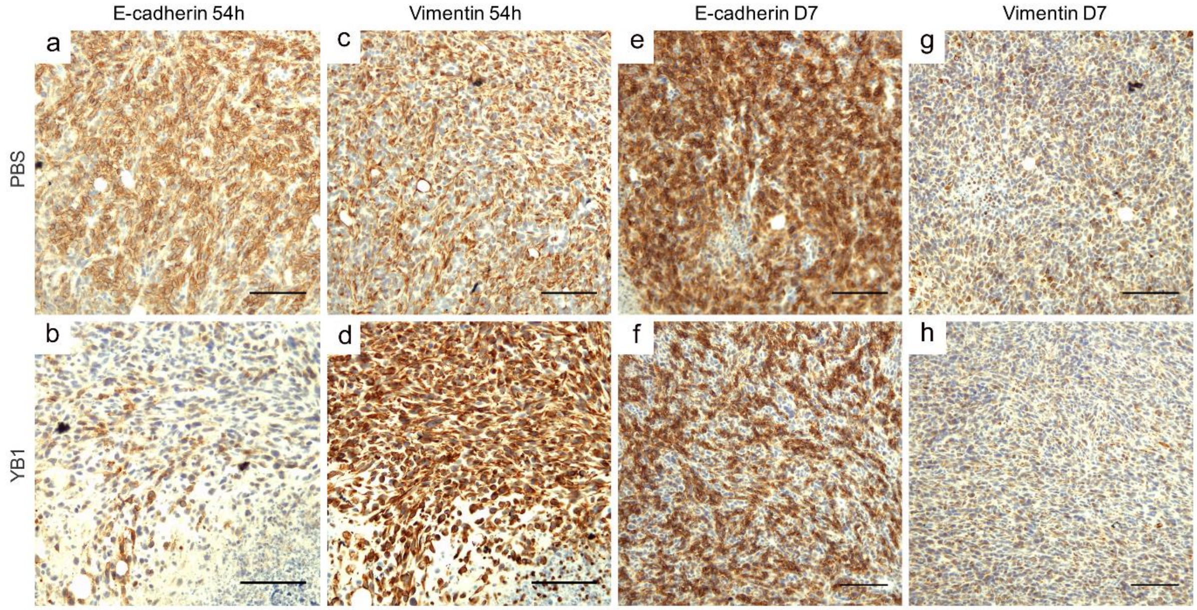

Supplementary Fig. 2 *Salmonella* YB1 treatment promotes EMT for a short time in the primary tumor. Primary tumors collected at 54 h (**a-d**) and on day 7 (D7) (**e-h**) from a 4T1-BALB/c orthotopic metastasis model stained with E-cadherin and vimentin as indicated. Scale bar, 100 µm. Displayed is one representative experiment of 2 independent experiments.

Supplementary Fig. 3

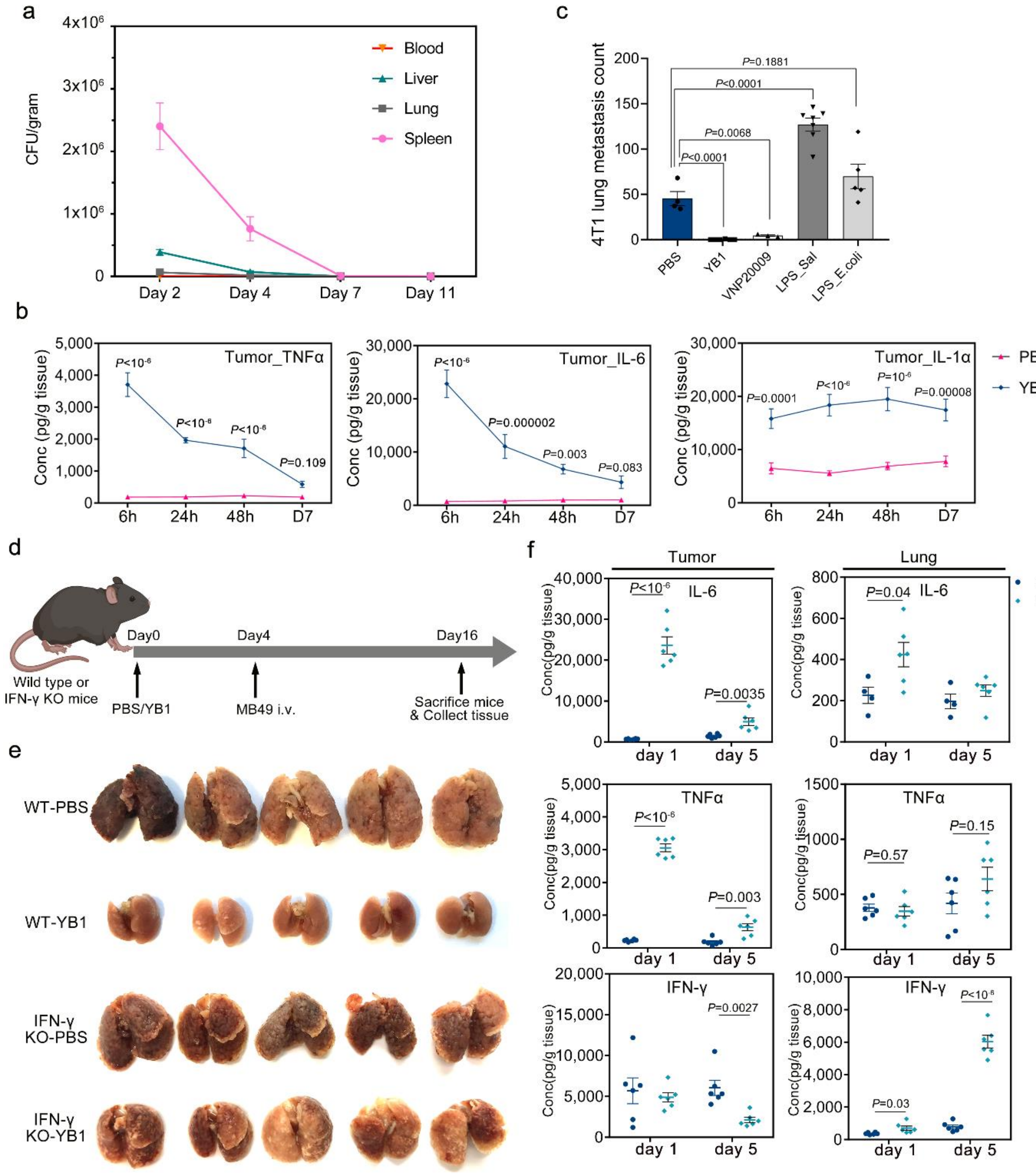

Supplementary Fig. 3 IFN-γ-dependent inflammation induced by *Salmonella* YB1 infection is indispensable to suppress cancer metastasis. a Distribution of *Salmonella* YB1 in blood, liver, lung, and spleen after i.v. injection into BALB/c mice (n=5 per each group). **b** Tumor-localized inflammatory cytokine responses after *Salmonella* YB1 treatment (n=6 per group). *P*-values were derived using two sided multiple t-tests. **c** Comparison of 4T1 lung metastases between different Salmonella strains and LPS. 2×10^7 CFU of YB1 (n=6), 2×10^7 CFU of VNP20009 (n=3), 10 ng of LPS from *Salmonella* (LPS_Sal, n=7), and 10 ng of LPS from *E. coli*

(LPS_E.coli, n=5) were injected i.v. to BALB/c mice at day 0, respectively. PBS treatment as a control (n=4). 4T1 cells were injected i.v. on day 5 to establish lung metastasis (*p*-values were derived using unpaired two-tailed t-tests). **d** Treatment procedures for the comparison of the anti-metastatic effect of YB1 on wild type C57BL/6J mice and IFN-γ knockout mice based on the experimental metastasis model established with MB49 cancer cells. **e** Comparison of the anti-metastatic activity of YB1 on wild type C57BL/6J mice and IFN-γ knockout mice (n=5 mice per group); 4% PFA-fixed lung tissues are shown. **f** Left column shows levels of cytokines IL-6, TNF-α, and IFN-γ in tumors collected on days 1 and 5 after YB1 treatment. The right column shows levels of cytokines IL-6, TNF-α, and IFN-γ in lungs collected on days 1 and 5 after YB1 treatment (n=6 mice per group, *p*-values were derived using multiple two-sided t-tests). All data are presented as mean values +/- s.e.m. Displayed is one representative experiment of 2 independent experiments. Source data are provided as a Source Data file.

Supplementary Fig. 4

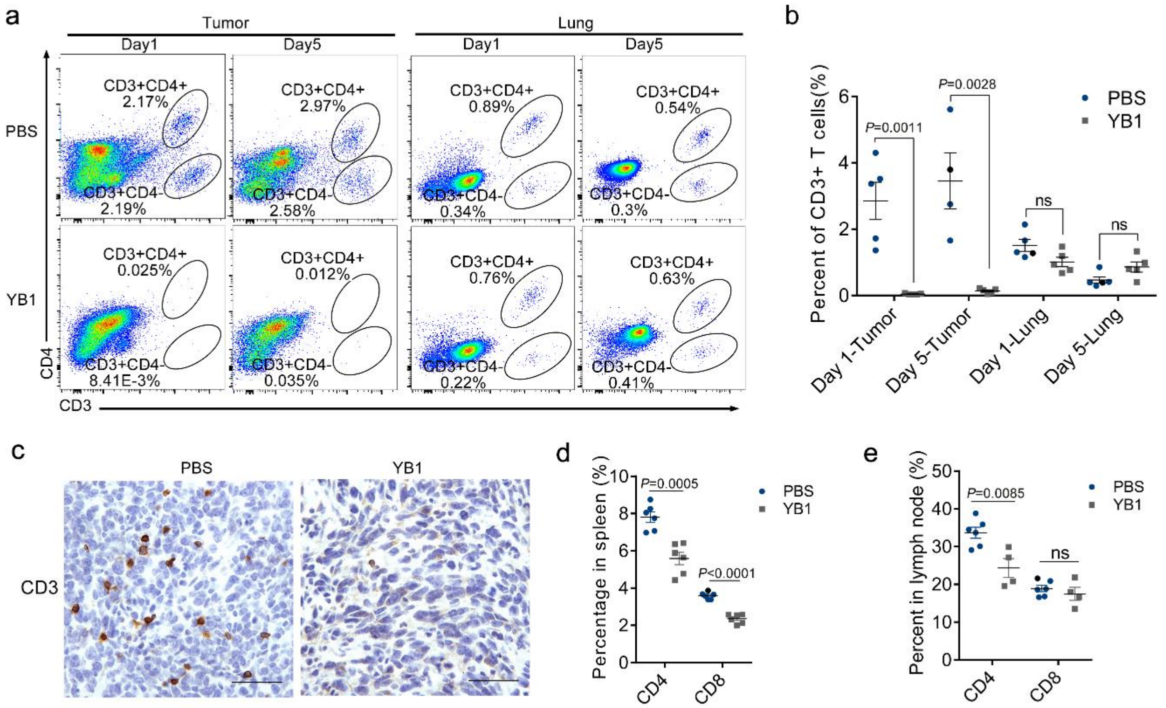

Supplementary Fig. 4 Reduction of T cells in tumor, spleen and tumor-draining lymph node after YB1 treatment. The 4T1-BALB/c orthotopic metastasis model was established and was treated with YB1 or PBS. **a** Representative flow cytometry plots of CD3+CD4+ and CD3+CD4- T cells in the tumor and lung of 4T1-BALB/c orthotopic metastasis model treated as indicated (n=5 mice per group). **b** Quantification of CD3+ T cells in tumor and lung tissues collected on day 1 and 5 after YB1 treatment, respectively (n=5 mice per group, *p*-values were derived using two tailed unpaired t tests). **c** Representative images of primary tumor sections stained with the anti-CD3 antibody (displayed is one representative experiment of 2 independent experiments). Tumors were collected on day 2 after YB1 treatment. Scale bar, 50μm. **d** Percentage of CD4+ and CD8+ T cells in spleen collected on day 5 after YB1 treatment was shown (n=6 mice per group, *p*-values were derived using two tailed unpaired t-tests). **e** Percentage of CD4+ and CD8+ T cells in tumor-draining lymph nodes collected on day 5 after YB1 treatment was shown (n=4 for YB1 group, n=6 for PBS group, *p*-values were derived using two tailed unpaired t-tests). All data are presented as mean values +/- s.e.m. Displayed is one representative experiment of 2 independent experiments. Source data are provided as a Source Data file.

Supplementary Fig. 5

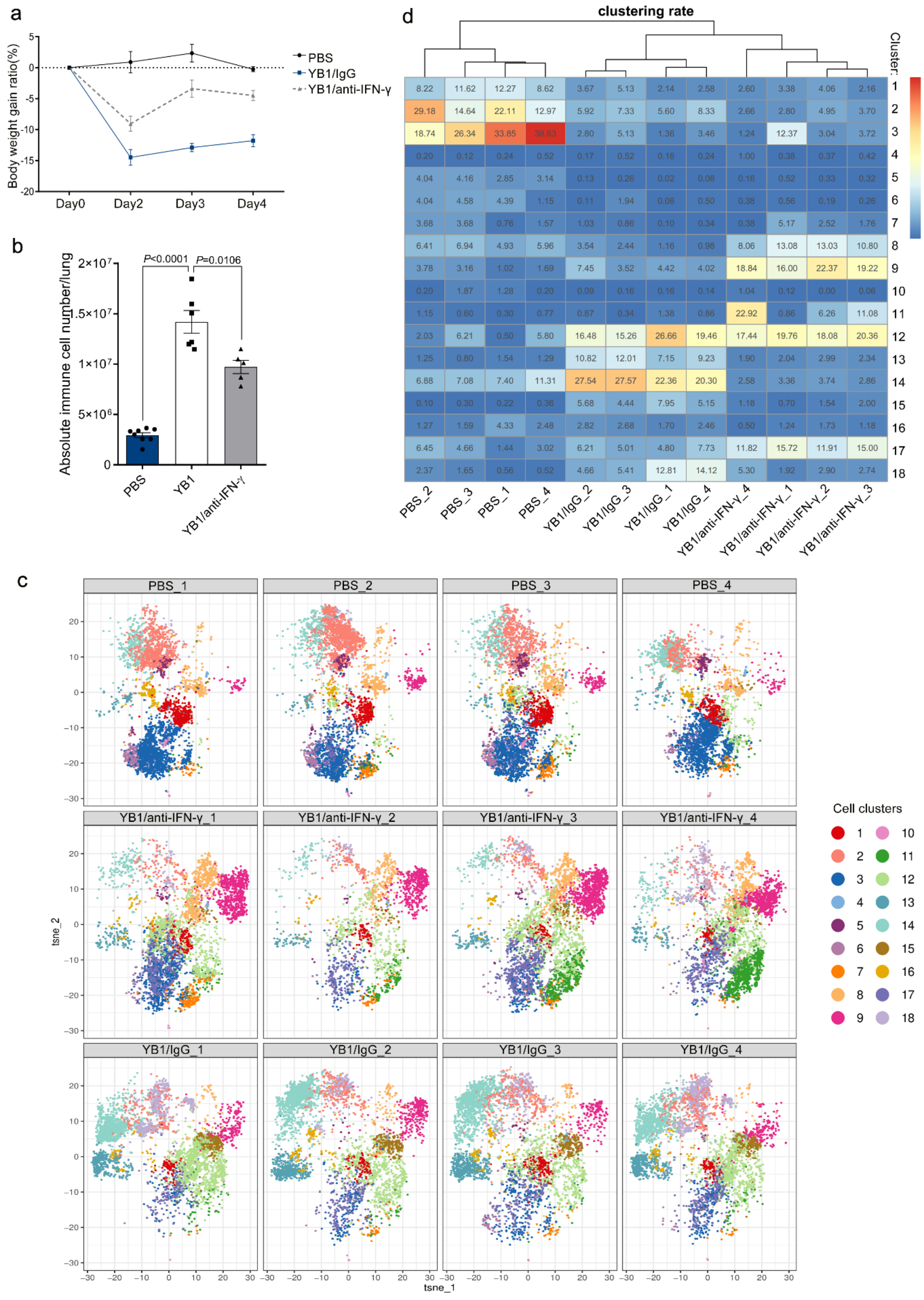

Supplementary Fig. 5 CyTOF analysis of lung infiltrating immune cells from NOD SCID mice to identify IFN-γ-dependent changes due to YB1. a-b NOD SCID mice were divided into three groups treated with PBS (n=8), YB1 plus IgG isotype (n=6), or YB1 plus IFN-γ depletion antibody (n=5). Body weight changes (**a**) and isolated lung infiltrating immune cells (**b**) of these mice were quantified before processing for CyTOF staining. *p*-values were derived using two-tailed unpaired t-tests . **c** t-SNE profiles of samples across three different treatments (n=4 for each group). **d** Clustering analysis of samples based on the results of t-SNE dimension reduction and PhenoGraph clustering analyses. All data are presented as mean values +/- s.e.m. Displayed is one representative experiment of 2 independent experiments. Source data are provided as a Source Data file.

Supplementary Fig. 6

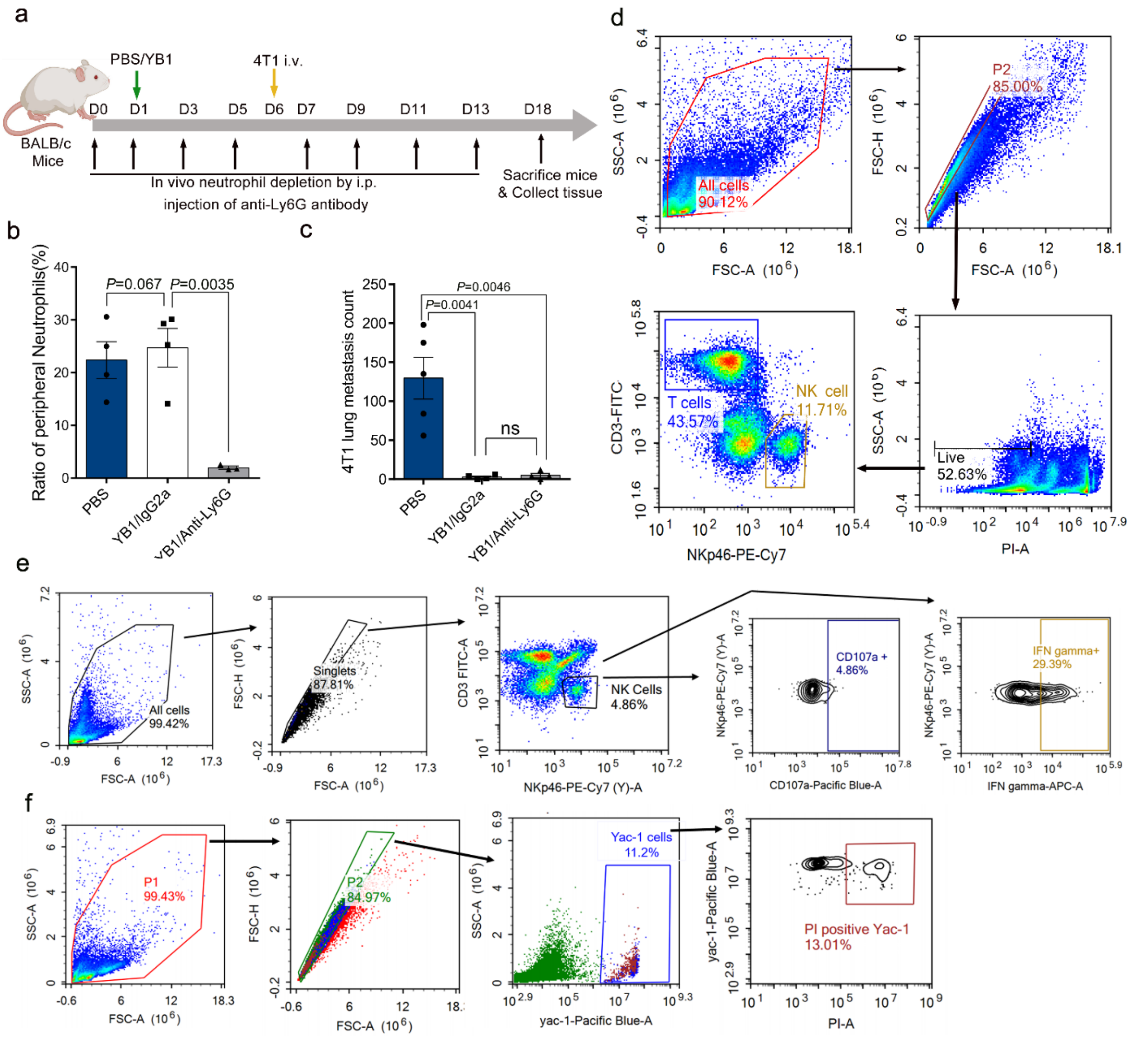

Supplementary Fig. 6 Neutrophils do not facilitate the suppression of metastasis after YB1 treatment. a Overall procedures for in vivo neutrophil depletion to confirm the role of

neutrophils in the anti-metastatic effect of YB1 treatment. Mice were treated with PBS, YB1 (YB1/IgG2a), or YB1 plus neutrophil depletion antibody (YB1/anti-Ly6G), respectively. Neutrophil depletion started 1 day before YB1 treatment and was maintained with i.p. injection of anti-Ly6G antibody every other day. **b** Neutrophil depletion efficiency was validated by flow cytometry analysis of peripheral neutrophils 7 days after the initiation of neutrophil depletion (n=3 for YB1/anti-Ly6G; n=4 for others). **c** Quantification of 4T1 lung metastases in the 4T1-BALB/c experimental metastasis model after the three different treatments (n=5 for PBS group, n=4 for others). Neutrophil depletion did not abolish the anti-metastatic effect of YB1 treatment. **d** General gating strategy of flow analysis for live cell analysis. Gates applied in every experiment: SSC-A/FSC-A gate was used to remove debris, FSC-A/FSC-H was used to gate singlets, then PI-positive gate was used to remove dead cells if not for intracellular staining. After this, cells were detected with fluorescent signals of relevant channels. This gating strategy was applied to Fig. 6b, f and Fig.7b, f, g. **e** Gating strategy of flow analysis for Fig. 6g-h and Fig. 7h-i. **f** Gating strategy of flow analysis for Fig. 6i and Fig. 7j. Boundaries were defined based on negative controls (isotype control staining). All *P*-values were derived using two-tailed unpaired t-tests. All data are presented as mean values +/- s.e.m. Displayed is one representative experiment of 2 independent experiments. Source data are provided as a Source Data file.

Supplementary Fig. 7

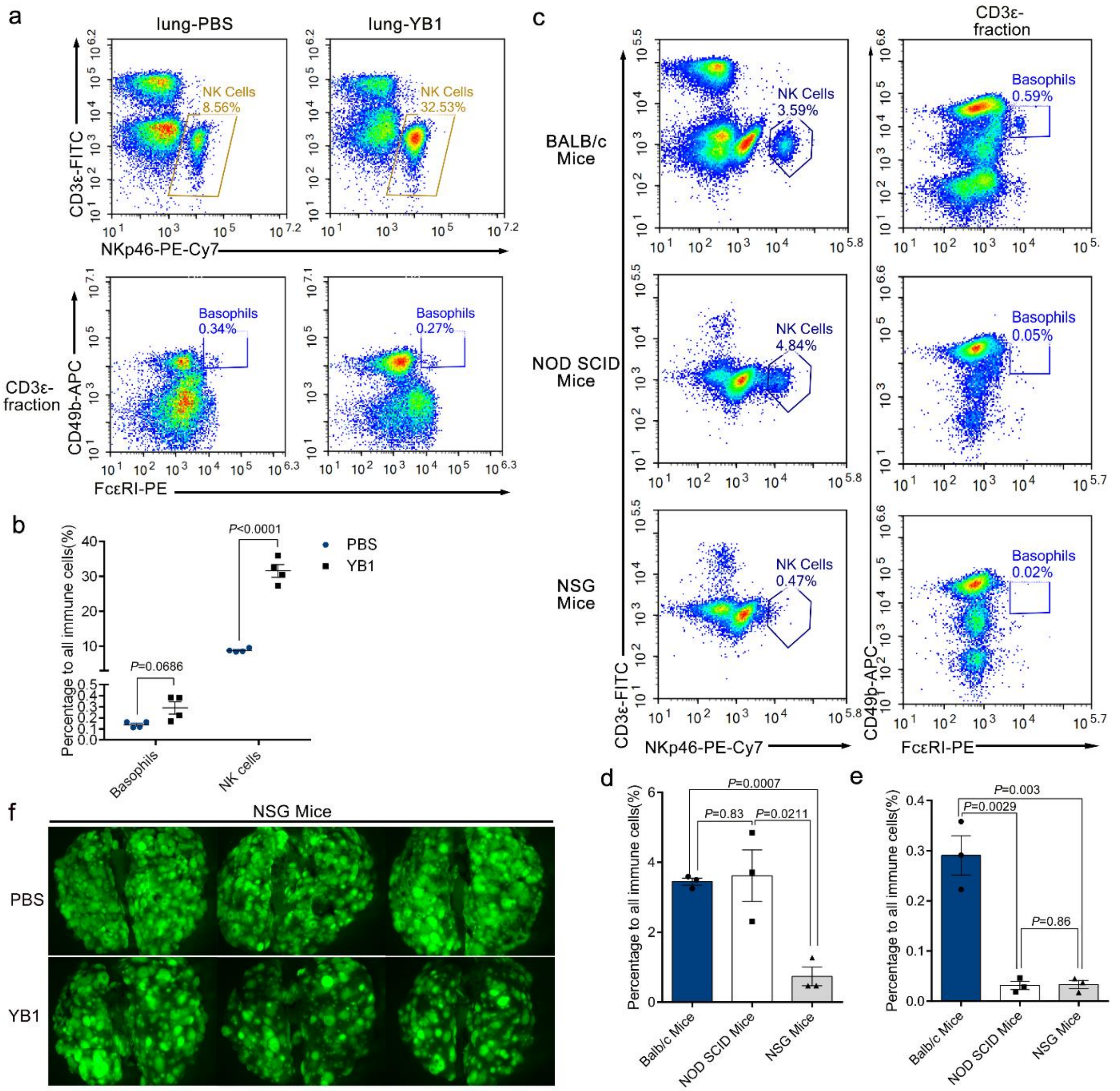

Supplementary Fig. 7 NK cells, rather than basophils, mediate the anti-metastatic effect of YB1. a Representative flow cytometry plots of CD3-NKp46+ NK cells and CD3-CD49b+ FcεRI + basophils in the lung from mice treated as indicated (n=4 mice per group). Lung tissue was collected 6 days after treatment. **b** Quantification of the ratios of CD3-NKp46+ NK cells and CD3-CD49b+ FcεRI + basophils mentioned in (**a**). **c** Representative flow cytometry plots of CD3-NKp46+ NK cells and CD3-CD49b+ FcεRI + basophils in BALB/c mice, NOD SCID mice, and NSG mice (n=3 mice per group). Blood samples were collected from three strains of mice for flow cytometry analysis. **d-e** Percentage of NK cells (**d**) and basophils (**e**) in different strains of mice as in (**c**). Each group have 3 mice. **f** Photos of 4T1-EGFP lung metastasis 12 days

after i.v. injection of 5×10^4 4T1-EGFP cells into NSG mice (n=3 per group). Overgrowth of 4T1-EGFP lung metastasis was found in all NSG mice treated either with PBS or YB1. All *p*-values were derived using two-tailed unpaired t-tests. All data are shown as the mean values +/- s.e.m. Displayed is one representative experiment of 2 independent experiments. Source data are provided as a Source Data file.

Supplementary Fig. 8

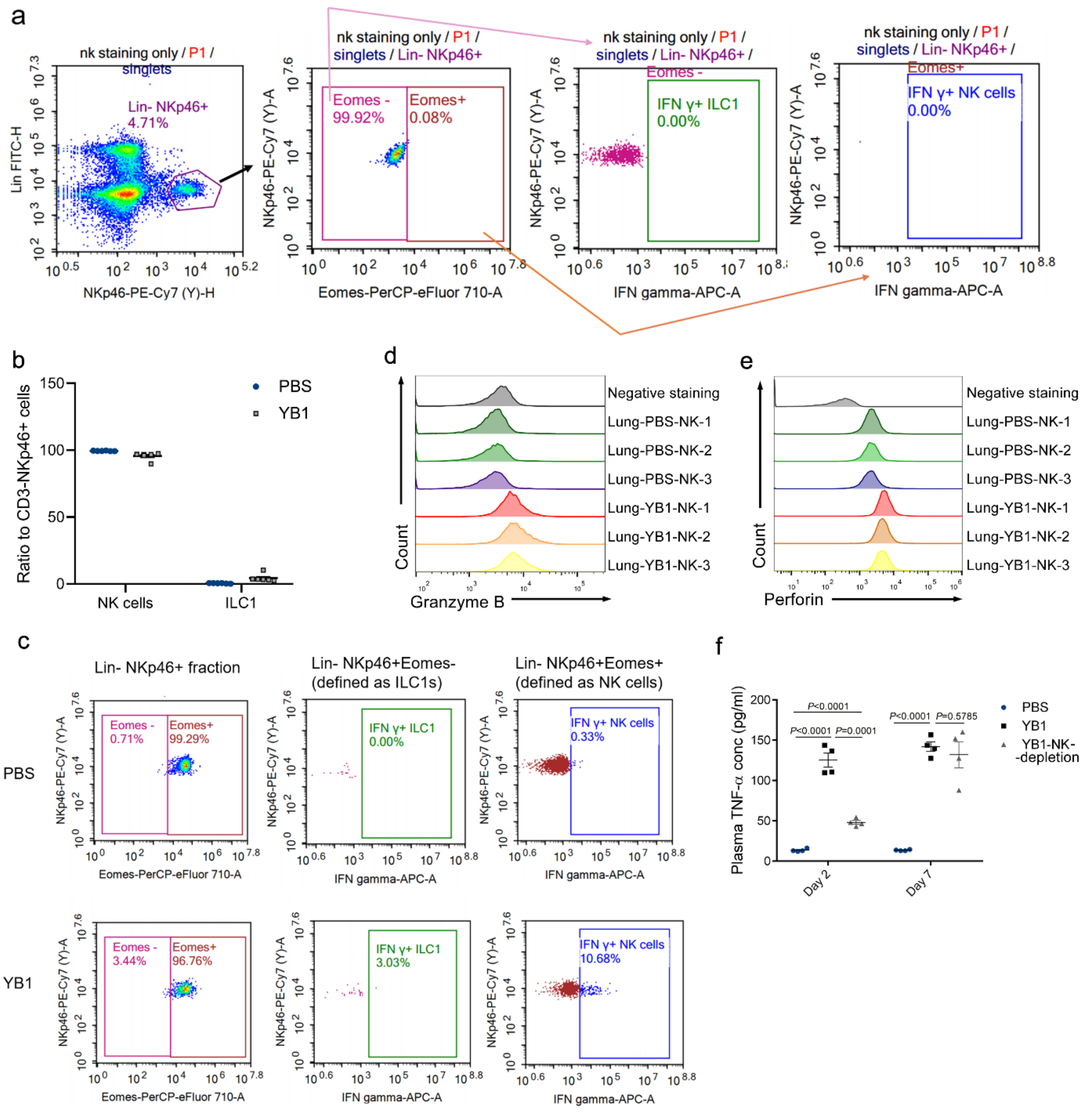

Supplementary Fig. 8 Lung infiltrating CD3-NKp46+ immune cells mainly are NK cells, rather than ILC1s. a Gating strategy to distinguish classical NK cells and ILC1s. Gating of Lin-NKp46+ was applied to singlets and further divided into two gates: Eomes- cells (ILC1s) and Eomes + cells (NK cells). IFN-γ signal was checked on ILC1s and NK cells. This sample

was only stained by anti-Lin antibody cocktail, anti-NKp46 antibody, isotype control of anti-Eomes antibody, and isotype control of anti-IFN-γ antibody. **b** Ratios of NK cells and ILC1 cells to total lung infiltrating Lin-NKp46+ cells. The graph was generated by combining two independent experiments (n=6 mice per group). **c** Representative flow cytometry dot plots illustrating the percentage of ILCs and NK cells from differentially treated mice, as well as their expression of IFN-γ after ex vivo co-culture with YAC-1 cells for 4 h. **d-e** BALB/c mice were divided into two groups and treated with PBS or 2×10^7 CFU of YB1. All mice were sacrificed 6 days after treatment and lung infiltrating immune cells were isolated for flow cytometric analysis of NK cells. Flow cytometric analysis of granzyme B and perforin on lung infiltrating NK cells across samples after co-culture with YAC-1 cells ex vivo for 5 h was performed (n=3 biological replicates). **f** BALB/c mice were divided into three groups and treated with PBS, YB1, or YB1 plus NK depletion antibody, respectively. NK cell depletion in vivo was started 1 day before YB1 treatment and maintained on days 1, 3, and 6 (n=4 mice per group). Plasma TNF-α concentration was measured on days 2, and 7 after YB1 and PBS treatment. **c-f** Displayed is one representative experiment of 2 independent experiments. All *p*-values were derived using two-tailed unpaired t-tests and data was shown as the mean values +/- s.e.m. Source data are provided as a Source Data file.

Supplementary Table 1. Antibodies and reagents for CyTOF

Isotope	Antibody	Clone	Distributor	Cat. number
141Pr	Ly-6G	1A8	Fluidigm	Cat# 3141008B
142Nd	CD11c	N418	Fluidigm	Cat# 3142003B
143Nd	IL-5	TRFK5	Fluidigm	Cat# 3143003B
144Nd	IL-2	JES6-5H4	Fluidigm	Cat# 3144002B
145Nd	CD69	H1.2F3	Fluidigm	Cat# 3145005B
146Nd	F4/80	BM8	Fluidigm	Cat# 3146008B
147Sm	CD45	30-F11	Fluidigm	Cat# 3147003B
148Nd	CD11b (Mac-1)	M1/70	Fluidigm	Cat# 3148003B
149Sm	CD19	6D5	Fluidigm	Cat# 3149002B
150Nd	CD44	IM7	Fluidigm	Cat# 3150018B
151Eu	CD25	PC-61.5.3	Bioxcell	Cat# BE0012
152Sm	CD3e	145-2C11	Fluidigm	Cat# 3152004B
153Eu	NKP46	29A1.4	Fluidigm	Cat# 3153006B
154Sm	TER-119	TER119	Fluidigm	Cat# 3154005B
155Gd	IL-10-purified	JES5-16E3	Biolegend	Cat# 505002
156Gd	IL-12-purified	C18.2	Biolegend	Cat# 511802
159Tb	CD279 (PD-1)	29F.1A12	Fluidigm	Cat# 3159024B
160Gd	CD62L (L-selectin)	MEL-14	Fluidigm	Cat# 3160008B
162Dy	TNFa	MP6-XT22	Fluidigm	Cat# 3162002B
163Dy	CD54 (ICAM-1)	YN1/1.7.4	Fluidigm	Cat# 3163020B
165Ho	IFNg	XMG1.2	Fluidigm	Cat# 3165003B
166Er	IL-4	11B11	Fluidigm	Cat# 3166003B
167Er	IL-6	MP5-20F3	Fluidigm	Cat# 3167003B
168Er	CD278/ICOS	C398.4A	Fluidigm	Cat# 3168024B

169Tm	TCRb	H57-597	Fluidigm	Cat# 3169002B
170Er	CD49b	HMa2	Fluidigm	Cat# 3170008B
171Yb	CD80	16-10A1	Fluidigm	Cat# 3171008B
172Yb	CD86	GL1	Fluidigm	Cat# 3172016B
173Yb	Ly6C-purified	HK1.4	Bioxcell	Cat# BE0284
174Yb	IL-17A	TC11-18H10.1	Fluidigm	Cat# 3174002B
175Lu	CD38	90	Fluidigm	Cat# 3175014B
176Yb	CD45R (B220)	RA3-6B2	Fluidigm	Cat# 3176002B
209Bi	I-A/I-E	M5/114.15.2	Fluidigm	Cat# 3209006B

Reagent		
Intercalator-103Rh	Fluidigm	Cat# 201103A
Intercalator-Ir	Fluidigm	Cat# 201192A
FcR Blocking Reagent, TruStain FcX™ (anti-mouse CD16/32) Antibody	Biolegend	Cat# 101320
Antibody Stabilizer based on PBS	Candor Biosciences	Cat# 131050
Bond-Breaker™ TCEP Solution， Neutral pH	Pierce	Cat# 77720
Calibration Beads, EQ(TM) Four Element	Fluidigm	Cat# 201078
Fix I Buffer (5X)	Fluidigm	Cat# 201065
Perm-S Buffer	Fluidigm	Cat# 201066
Cell Staining Buffer	Fluidigm	Cat# 201068
Maxpar® Water	Fluidigm	Cat# 201069
Maxpar X8 Multimetal labeling kit	Fluidigm	Cat# 201300
Lanthanide (III) metal isotopes as chloride salts	Fluidigm	N/A

Amicon Ultra-0.5 Centrifugal Filter Unit with Ultracel-3 membrane	Millipore	Cat# UFC500396
Amicon Ultra-0.5 Centrifugal Filter Unit with Ultracel-50 membrane	Millipore	Cat# UFC505096

Supplementary Table 2. Antibodies used for flow cytometry analysis and immunohistochemistry

Antigen	Fluorescent conjugate	Vendor	Cat. number	Clone name	Dilution
CD3	PE	BioLegend	100206	17A1	1:150
CD4	Alexa Fluor 488	BioLegend	100423	GK1.5	1:1000
CD8a	PE	BioLegend	100708	53-6.7	1:100
CD3	FITC	eBioscience	11-0032-82	17A2	1:200
Gr-1	Brilliant Violet 421	BioLegend	108433	RB6-8C5	1:50
Ly-6G	PE-Cy7	BioLegend	127618	1A8	1:200
CD49b	APC	BioLegend	108910	DX5	1:100
NKp46	PE-Cy7	eBioscience	25-3351-82	29A1.4	1:50
NKp46	PE	eBioscience	12-3351-80	29A1.4	1:50
CD45	PE	BioLegend	103106	30-F11	1:100
IFN-γ	APC	eBioscience	17-7311-81	XMG1.2	1:100
CD11b	eFluor 450	eBioscience	48-0112-80	M1/70	1:100
CD27	Super Bright 600	eBioscience	63-0271-80	LG.7F9	1:50
CD11c	Alexa Fluor 700	eBioscience	56-0114-80	N418	1:50
NKG2D	APC	Thermo Fisher Scientific	17-5882-81	CX5	1:100
CD38	BV711	BD Biosciences	740697	90/CD38	1:100
CD107a	V450	BD Biosciences	560648	1D4B	1:100
Perforin	APC	Thermo Fisher Scientific	17-9392-80	eBioOMAK-D	1:100
Granzyme B	eFluor 450	Thermo Fisher Scientific	48-8898-80	NGZB	1:100
Lineage Cocktail	FITC	BioLegend	133301	145-2C11; RB6-8C5; RA3-6B2; Ter-119; M1/70;	1:10
FceR1 alpha	PE	Invitrogen	12-5898-81	MAR-1	1:100
Eomes	PerCP-eFluor 710	Invitrogen	46-4875-80	Dan11mag	1:100
CD3	No conjugate	abcam	Ab16669	SP7	1:150
E-cadherin	No conjugate	Cell Signaling	#3195	24E10	1:400
Vimentin	No conjugate	Cell Signaling	#5741	D21H3	1:200
