# Supplementary material for: IFN-γ-dependent NK cell activation is essential to metastasis suppression by engineered Salmonella
Source: Nat Commun. 2021 May 5;12:2537. doi: 10.1038/s41467-021-22755-3 (PMC8099885; doi:10.1038/s41467-021-22755-3)
Supplement: Supplementary file 3 — Reporting Summary [file 41467_2021_22755_MOESM3_ESM.pdf]

nature research

Corresponding author(s): Jiandong Huang

Last updated by author(s): Feb 22, 2021

Reporting Summary

Nature Research wishes to improve the reproducibility of the work that we publish. This form provides structure for consistency and transparency in reporting. For further information on Nature Research policies, see Authors & Referees and the Editorial Policy Checklist .

Statistics

For all statistical analyses, confirm that the following items are present in the figure legend, table legend, main text, or Methods section.

n/a	Confirmed	
☐	☒	The exact sample size (*n*) for each experimental group/condition, given as a discrete number and unit of measurement
☐	☒	A statement on whether measurements were taken from distinct samples or whether the same sample was measured repeatedly
☐	☒	The statistical test(s) used AND whether they are one- or two-sided<br>*Only common tests should be described solely by name; describe more complex techniques in the Methods section.*
☒	☐	A description of all covariates tested
☒	☐	A description of any assumptions or corrections, such as tests of normality and adjustment for multiple comparisons
☐	☒	A full description of the statistical parameters including central tendency (e.g. means) or other basic estimates (e.g. regression coefficient) AND variation (e.g. standard deviation) or associated estimates of uncertainty (e.g. confidence intervals)
☐	☒	For null hypothesis testing, the test statistic (e.g. *F*, *t*, *r*) with confidence intervals, effect sizes, degrees of freedom and *P* value noted<br>*Give P values as exact values whenever suitable.*
☒	☐	For Bayesian analysis, information on the choice of priors and Markov chain Monte Carlo settings
☒	☐	For hierarchical and complex designs, identification of the appropriate level for tests and full reporting of outcomes
☒	☐	Estimates of effect sizes (e.g. Cohen's *d*, Pearson's *r*), indicating how they were calculated

Our web collection on statistics for biologists contains articles on many of the points above.

Software and code

Policy information about availability of computer code

Data collection	Luciferase live imaging: Images were taken with the PE IVIS Spectrum in vivo imaging system;<br>Mass cytometry: The samples were acquired on a CyTOF2 (Fluidigm) at an event rate of <500 events/second.<br>Flow Cytometry: BD LSR Fortessa Analyzer, ACEA Novocyte Flow Cytometer were used for the flow cytometric data acquisition.
Data analysis	Luciferase live imaging: Data was analyzed with Living image 4.0 software;<br>Mass cytometry: After the acquisition, the data were normalized using bead-based normalization in the CyTOF software (Version 6.7.1014, Fluidigm). Mass cytometry data were normalized to EQ 4-element bead signal (Lot P15K0802, Passport EQ 4_P13H2302) in 100s interval windows using the normalization function in the CyTOF software (Version 6.7.1014, Fluidigm). Mass tag barcodes were also resolved with a doublet filtering scheme using Debarcoder in the CyTOF software (Version 6.7.1014, Fluidigm). Live immune cells were manually gated in FlowJo by event length, live/dead discrimination, and the desired expression of CD45. Data were then exported for downstream analysis and transformed with a coefficient of 5 with method cytofAsinh. For most downstream analyses, the individual sample data were subsampled to 5000 events (or all events were sampled if total cell number was less than 5,000).All samples had at least 2000 events. t-Distributed Stochastic Neighbor Embedding (t-SNE) dimension reduction and PhenoGraph clustering analyses were performed using the tool cytofkit run in R.<br>Flow Cytometry: FlowJo_v10.6.2, and NovoExpress_v1.4.1 were used for the flow cytometric analysis.<br>Graphpad Prism 8.0.1 software was used to construct all graphs and calculate statistical significance.

For manuscripts utilizing custom algorithms or software that are central to the research but not yet described in published literature, software must be made available to editors/reviewers. We strongly encourage code deposition in a community repository (e.g. GitHub). See the Nature Research guidelines for submitting code & software for further information.

Data

Policy information about availability of data

All manuscripts must include a data availability statement. This statement should provide the following information, where applicable:

- Accession codes, unique identifiers, or web links for publicly available datasets
- A list of figures that have associated raw data
- A description of any restrictions on data availability

All data that supporting the findings of this study are available in the Article and Supplementary Information or available from the corresponding author upon reasonable request.

Field-specific reporting

Please select the one below that is the best fit for your research. If you are not sure, read the appropriate sections before making your selection.

☒ Life sciences ☐ Behavioural & social sciences ☐ Ecological, evolutionary & environmental sciences

For a reference copy of the document with all sections, see nature.com/documents/nr-reporting-summary-flat.pdf

Life sciences study design

All studies must disclose on these points even when the disclosure is negative.

Sample size	Sample sizes were based on empirical data from pilot experiments and similar published studies(at least 4 animals were needed for each condition and two independent experiments were guaranteed for all data; At least 3 biological repeats were needed for flow cytometry analysis). For all statistics analysis, 95% confidence interval is used:<br>1. Loo, J. M. et al. Extracellular metabolic energetics can promote cancer progression. Cell 160, 393-406, doi:10.1016/j.cell.2014.12.018 (2015).<br>2 Gunderson, A. J. et al. TGFbeta suppresses CD8(+) T cell expression of CXCR3 and tumor trafficking. Nat Commun 11, 1749, doi:10.1038/s41467-020-15404-8 (2020).<br>3. Hsu, J. et al. Contribution of NK cells to immunotherapy mediated by PD-1/PD-L1 blockade. J Clin Invest 128, 4654-4668, doi:10.1172/JCI99317 (2018).
Data exclusions	No data were excluded.
Replication	All attempts at replication were successful. All data in the paper was successfully replicated in at least two independent experiments performed under identical conditions. Cell cultures were regularly checked for mycoplasma. Key results were confirmed in different models. For example, the anti-metastasis effect of YB1 was confirmed in mutiple syngeneic tumor models. The effect of IFN-γ was validated by in vivo antibody-dependent depletion and IFN-γ knockout mice.
Randomization	For all mouse experiments, we always randomly distribute mice into different groups and never excluded any data;
Blinding	The investigators were blinded to group allocation during data collection and data analysis for all experiments. Usually data collection and analysis were finished by different investigators.

Behavioural & social sciences study design

All studies must disclose on these points even when the disclosure is negative.

Study description	*Briefly describe the study type including whether data are quantitative, qualitative, or mixed-methods (e.g. qualitative cross-sectional, quantitative experimental, mixed-methods case study).*
Research sample	*State the research sample (e.g. Harvard university undergraduates, villagers in rural India) and provide relevant demographic information (e.g. age, sex) and indicate whether the sample is representative. Provide a rationale for the study sample chosen. For studies involving existing datasets, please describe the dataset and source.*
Sampling strategy	*Describe the sampling procedure (e.g. random, snowball, stratified, convenience). Describe the statistical methods that were used to predetermine sample size OR if no sample-size calculation was performed, describe how sample sizes were chosen and provide a rationale for why these sample sizes are sufficient. For qualitative data, please indicate whether data saturation was considered, and what criteria were used to decide that no further sampling was needed.*
Data collection	*Provide details about the data collection procedure, including the instruments or devices used to record the data (e.g. pen and paper, computer, eye tracker, video or audio equipment) whether anyone was present besides the participant(s) and the researcher, and whether the researcher was blind to experimental condition and/or the study hypothesis during data collection.*

Timing	*Indicate the start and stop dates of data collection. If there is a gap between collection periods, state the dates for each sample cohort.*
Data exclusions	*If no data were excluded from the analyses, state so OR if data were excluded, provide the exact number of exclusions and the rationale behind them, indicating whether exclusion criteria were pre-established.*
Non-participation	*State how many participants dropped out/declined participation and the reason(s) given OR provide response rate OR state that no participants dropped out/declined participation.*
Randomization	*If participants were not allocated into experimental groups, state so OR describe how participants were allocated to groups, and if allocation was not random, describe how covariates were controlled.*

Ecological, evolutionary & environmental sciences study design

All studies must disclose on these points even when the disclosure is negative.

Study description	*Briefly describe the study. For quantitative data include treatment factors and interactions, design structure (e.g. factorial, nested, hierarchical), nature and number of experimental units and replicates.*
Research sample	*Describe the research sample (e.g. a group of tagged Passer domesticus, all Stenocereus thurberi within Organ Pipe Cactus National Monument), and provide a rationale for the sample choice. When relevant, describe the organism taxa, source, sex, age range and any manipulations. State what population the sample is meant to represent when applicable. For studies involving existing datasets, describe the data and its source.*
Sampling strategy	*Note the sampling procedure. Describe the statistical methods that were used to predetermine sample size OR if no sample-size calculation was performed, describe how sample sizes were chosen and provide a rationale for why these sample sizes are sufficient.*
Data collection	*Describe the data collection procedure, including who recorded the data and how.*
Timing and spatial scale	*Indicate the start and stop dates of data collection, noting the frequency and periodicity of sampling and providing a rationale for these choices. If there is a gap between collection periods, state the dates for each sample cohort. Specify the spatial scale from which the data are taken*
Data exclusions	*If no data were excluded from the analyses, state so OR if data were excluded, describe the exclusions and the rationale behind them, indicating whether exclusion criteria were pre-established.*
Reproducibility	*Describe the measures taken to verify the reproducibility of experimental findings. For each experiment, note whether any attempts to repeat the experiment failed OR state that all attempts to repeat the experiment were successful.*
Randomization	*Describe how samples/organisms/participants were allocated into groups. If allocation was not random, describe how covariates were controlled. If this is not relevant to your study, explain why.*
Blinding	*Describe the extent of blinding used during data acquisition and analysis. If blinding was not possible, describe why OR explain why blinding was not relevant to your study.*

Did the study involve field work? ☐ Yes ☐ No

Field work, collection and transport

Field conditions	*Describe the study conditions for field work, providing relevant parameters (e.g. temperature, rainfall).*
Location	*State the location of the sampling or experiment, providing relevant parameters (e.g. latitude and longitude, elevation, water depth).*
Access and import/export	*Describe the efforts you have made to access habitats and to collect and import/export your samples in a responsible manner and in compliance with local, national and international laws, noting any permits that were obtained (give the name of the issuing authority, the date of issue, and any identifying information).*
Disturbance	*Describe any disturbance caused by the study and how it was minimized.*

Reporting for specific materials, systems and methods

We require information from authors about some types of materials, experimental systems and methods used in many studies. Here, indicate whether each material, system or method listed is relevant to your study. If you are not sure if a list item applies to your research, read the appropriate section before selecting a response.

Materials & experimental systems

n/a	Involved in the study
☐	☒ Antibodies
☐	☒ Eukaryotic cell lines
☒	☐ Palaeontology
☐	☒ Animals and other organisms
☒	☐ Human research participants
☒	☐ Clinical data

Methods

n/a	Involved in the study
☒	☐ ChIP-seq
☐	☒ Flow cytometry
☒	☐ MRI-based neuroimaging

Antibodies

Antibodies used

Antibodies used in flow analysis and immunohistochemistry (Supplementary table 2):
Antigen; Fluorescent conjugate ; Vendor ; Cat.no ; Clone name ; Dilution
CD3 ; PE ; BioLegend; 100206; 17A1 ; 1:150
CD4; Alexa Fluor 488 ; BioLegend ; 100423 ; GK1.5 ; 1:1000
CD8a; PE; BioLegend ; 100708 ; 53-6.7 ; 1:100
CD3 ; FITC; eBioscience ; 11-0032-82 ; 17A2 ; 1:200
Gr-1; Brilliant Violet 421 ; BioLegend; 108433; RB6-8C5; 1:50
Ly-6G; PE-Cy7 ; BioLegend ; 127618 ; 1A8 ; 1:200
CD49b ; APC ; BioLegend ; 108910; DX5; 1:100
NKp46 ; PE-Cy7; eBioscience ; 25-3351-82 ; 29A1.4; 1:50
NKp46 ; PE ; eBioscience ; 12-3351-80; 29A1.4 ; 1:50
CD45; PE; BioLegend ; 103106; 30-F11; 1:100
IFN-γ ; APC ; eBioscience ; 17-7311-81 ; XMG1.2 ; 1:100
CD11b ; eFluor 450; eBioscience; 48-0112-80; M1/70 ; 1:100
CD27; Super Bright 600 ; eBioscience; 63-0271-80; LG.7F9 ; 1:50
CD11c; Alexa Fluor 700; eBioscience ; 56-0114-80; N418; 1:50
NKG2D ; APC; Thermo Fisher Scientific ; 17-5882-81; CX5 ; 1:100
CD38 ; BV711 ; BD Biosciences ; 740697; 90/CD38 ; 1:100
CD107a; V450 ; BD Biosciences ; 560648; 1D4B ; 1:100
Perforin ; APC; Thermo Fisher Scientific ; 17-9392-80; eBioOMAK-D ; 1:100
Granzyme B; eFluor 450 ; Thermo Fisher Scientific ; 48-8898-80; NGZB; 1:100
Lineage Cocktail; FITC ; BioLegend ; 133301 ; (145-2C11; RB6-8C5; RA3-6B2; Ter-119; M1/70); 1:10
FceR1 alpha; PE; Invitrogen ; 12-5898-81 ; MAR-1; 1:100
Eomes ; PerCP-eFluor 710; Invitrogen ; 46-4875-80 ; Dan11mag; 1:100
CD3; No conjugate; abcam ; Ab16669; SP7 ; 1:150
E-cadherin; No conjugate; Cell Signaling; #3195; 24E10 ; 1:400
Vimentin; No conjugate ; Cell Signaling ; #5741 ; D21H3; 1:200

Antibodies used in vivo: anti-Asialo GM-1 (#146002; clone Poly21460; BioLegend); polyclonal rabbit IgG(#BE0095; polyclone; BioXcell); anti-Ly6G antibody (#BP0075-1; clone 1A8; BioXCell); Rat IgG2a isotype control (#BP0089; clone 2A3; BioXCell); anti-TNF-α (#16-7423-81; clone TN3-19.12; eBioscience); anti-IFN-γ (#16-7411-85; clone H22; eBioscience); Armenian Hamster IgG Isotype Control (#16-4888-38; eBio299Arm; eBioscience). The dosage of each antibody used in vivo was addressed in Methods section in the manuscript.
Antibodies used for CyTOF analysis are listed in Supplementary table 1. All dilutions for these antibodies are 1:100.

Validation

All antibodies were validated by the manufacturers

Eukaryotic cell lines

Policy information about cell lines

Cell line source(s)

Mouse 4T1 breast cancer cells, CT26 colon cancer cells and B16F10 melanoma cells were purchased from ATCC; Murine YAC-1 cells were kindly provided by Stem Cell Bank, Chinese Academy of Sciences; L1210-GFP cells were generously provided by Dr. Gaoliang Ouyang, Xiamen University; MB49 cancer cells were generously provided by Dr. LIU Chenli, Shenzhen Institute of Synthetic Biology.

Authentication

ATCC and Stem Cell Bank, Chinese Academy of Science validate their cell lines; L1210-GFP and MB49 cells were validated by the parent labs. No authentication was performed for these cell lines by ourselves.

Mycoplasma contamination

All cells are routinely screened for mycoplasma at Core Facility, LKS Faculty of Medicine, The University of Hong Kong. All cell lines tested were negative for mycoplasma contamination.

Commonly misidentified lines (See ICLAC register) | No commonly misidentified cell lines were used.

Palaeontology

Specimen provenance | *Provide provenance information for specimens and describe permits that were obtained for the work (including the name of the issuing authority, the date of issue, and any identifying information).*

Specimen deposition | *Indicate where the specimens have been deposited to permit free access by other researchers.*

Dating methods | *If new dates are provided, describe how they were obtained (e.g. collection, storage, sample pretreatment and measurement), where they were obtained (i.e. lab name), the calibration program and the protocol for quality assurance OR state that no new dates are provided.*

☐ Tick this box to confirm that the raw and calibrated dates are available in the paper or in Supplementary Information.

Animals and other organisms

Policy information about studies involving animals; ARRIVE guidelines recommended for reporting animal research

Laboratory animals | The 6- to 8-week-old female BALB/c, NOD.Cg-PrkdcscidIl2rgtm1Wjl/SzJ (NSG), and NOD.CB17-Prkdcscid/J (NOD SCID) mice were purchased from the Laboratory Animal Unit of The University of Hong Kong. The 6- to 8-week-old female C57BL/6J mice were purchased from the Laboratory Animal Unit of Shenzhen Institutes of Advanced Technology Chinese Academy of Sciences. The IFN-γ knockout mice (JAX stock #002287) were purchased from The Jackson Laboratory. Mice were maintained in a 12 h light/12 h dark cycle at ~23 °C and 40% relative humidity with food and water ad libitum.

Wild animals | This study didn't involve wild animals

Field-collected samples | This study did not involve samples collected from the field

Ethics oversight | All mice experiments conducted in Hong Kong were approved by the Committee on the Use of Live Animals in Teaching and Research (CULATR) of The University of Hong Kong. All animal experiments conducted in Shenzhen complied with protocols approved by the Shenzhen Institutes of Advanced Technology Chinese Academy of Sciences Committee on Animal Care.

Note that full information on the approval of the study protocol must also be provided in the manuscript.

Human research participants

Policy information about studies involving human research participants

Population characteristics | *Describe the covariate-relevant population characteristics of the human research participants (e.g. age, gender, genotypic information, past and current diagnosis and treatment categories). If you filled out the behavioural & social sciences study design questions and have nothing to add here, write "See above."*

Recruitment | *Describe how participants were recruited. Outline any potential self-selection bias or other biases that may be present and how these are likely to impact results.*

Ethics oversight | *Identify the organization(s) that approved the study protocol.*

Note that full information on the approval of the study protocol must also be provided in the manuscript.

Clinical data

Policy information about clinical studies

All manuscripts should comply with the ICMJEguidelines for publication of clinical research and a completedCONSORT checklist must be included with all submissions.

Clinical trial registration | *Provide the trial registration number from ClinicalTrials.gov or an equivalent agency.*

Study protocol | *Note where the full trial protocol can be accessed OR if not available, explain why.*

Data collection | *Describe the settings and locales of data collection, noting the time periods of recruitment and data collection.*

Outcomes | *Describe how you pre-defined primary and secondary outcome measures and how you assessed these measures.*

ChIP-seq

Data deposition

☐ Confirm that both raw and final processed data have been deposited in a public database such as GEO.

☐ Confirm that you have deposited or provided access to graph files (e.g. BED files) for the called peaks.

Data access links<br>*May remain private before publication.*	*For "Initial submission" or "Revised version" documents, provide reviewer access links. For your "Final submission" document, provide a link to the deposited data.*
Files in database submission	*Provide a list of all files available in the database submission.*
Genome browser session<br>(e.g. UCSC)	*Provide a link to an anonymized genome browser session for "Initial submission" and "Revised version" documents only, to enable peer review. Write "no longer applicable" for "Final submission" documents.*

Methodology

Replicates	*Describe the experimental replicates, specifying number, type and replicate agreement.*
Sequencing depth	*Describe the sequencing depth for each experiment, providing the total number of reads, uniquely mapped reads, length of reads and whether they were paired- or single-end.*
Antibodies	*Describe the antibodies used for the ChIP-seq experiments; as applicable, provide supplier name, catalog number, clone name, and lot number.*
Peak calling parameters	*Specify the command line program and parameters used for read mapping and peak calling, including the ChIP, control and index files used.*
Data quality	*Describe the methods used to ensure data quality in full detail, including how many peaks are at FDR 5% and above 5-fold enrichment.*
Software	*Describe the software used to collect and analyze the ChIP-seq data. For custom code that has been deposited into a community repository, provide accession details.*

Flow Cytometry

Plots

Confirm that:

☒ The axis labels state the marker and fluorochrome used (e.g. CD4-FITC).

☒ The axis scales are clearly visible. Include numbers along axes only for bottom left plot of group (a 'group' is an analysis of identical markers).

☒ All plots are contour plots with outliers or pseudocolor plots.

☒ A numerical value for number of cells or percentage (with statistics) is provided.

Methodology

Sample preparation	Primarily isolated mouse immune cells and YAC-1 cancer cell line were applied to flow analysis in this study. For cell surface markers detection, immune cells were stained with antibodies for 30 min on ice and washed twice with 1% BSA/PBS before flow cytometric analysis. For intracellular staining, immune cells were induced ex vivo with PMA/ionomycin (or YAC-1 cancer cell) supplemented with brefeldin A for 5 h. After culture, cell mixtures were collected and stained for cell surface markers. Following fixation and permeabilization of cells (BD, catalog no. 554714), cells were stained with intracellular staining antibodies on ice for 30 min and washed twice before flow cytometric analysis.
Instrument	BD LSR Fortessa Analyzer, ACEA Novocyte Flow Cytometer were used to collect data
Software	FlowJo_v10.6.2, and NovoExpress_v1.4.1 were used for the flow cytometric analysis.
Cell population abundance	The stop gating setting for most flow cytometry analysis: the number of singlets should be more than 20000 events;<br>The stop gating setting for flow cytometry-based killing assay: YAC-1 target cells are more than 4000 events (only 10000 cells were seeded into each well); The abundance of relevant cell populations were labeled with percentage in raw PDF data and quantified in main figures.

Gating strategy	Gates applied in every experiments: SSC-A/FSC-A gate was used to remove debris, FSC-A/FSC-H was used to gate singlets, then PI positive gate was used to remove dead cells. After this, cells were detected with fluorescent signals of relevant channels. Boundaries were defined based on negative controls (isotype control stainings). The gating strategies were addressed in Supplementary Fig. 6d-f.

☒ Tick this box to confirm that a figure exemplifying the gating strategy is provided in the Supplementary Information.

Magnetic resonance imaging

Experimental design

Design type	*Indicate task or resting state; event-related or block design.*
Design specifications	*Specify the number of blocks, trials or experimental units per session and/or subject, and specify the length of each trial or block (if trials are blocked) and interval between trials.*
Behavioral performance measures	*State number and/or type of variables recorded (e.g. correct button press, response time) and what statistics were used to establish that the subjects were performing the task as expected (e.g. mean, range, and/or standard deviation across subjects).*

Acquisition

Imaging type(s)	*Specify: functional, structural, diffusion, perfusion.*
Field strength	*Specify in Tesla*
Sequence & imaging parameters	*Specify the pulse sequence type (gradient echo, spin echo, etc.), imaging type (EPI, spiral, etc.), field of view, matrix size, slice thickness, orientation and TE/TR/flip angle.*
Area of acquisition	*State whether a whole brain scan was used OR define the area of acquisition, describing how the region was determined.*

Diffusion MRI ☐ Used ☐ Not used

Preprocessing

Preprocessing software	*Provide detail on software version and revision number and on specific parameters (model/functions, brain extraction, segmentation, smoothing kernel size, etc.).*
Normalization	*If data were normalized/standardized, describe the approach(es): specify linear or non-linear and define image types used for transformation OR indicate that data were not normalized and explain rationale for lack of normalization.*
Normalization template	*Describe the template used for normalization/transformation, specifying subject space or group standardized space (e.g. original Talairach, MNI305, ICBM152) OR indicate that the data were not normalized.*
Noise and artifact removal	*Describe your procedure(s) for artifact and structured noise removal, specifying motion parameters, tissue signals and physiological signals (heart rate, respiration).*
Volume censoring	*Define your software and/or method and criteria for volume censoring, and state the extent of such censoring.*

Statistical modeling & inference

Model type and settings	*Specify type (mass univariate, multivariate, RSA, predictive, etc.) and describe essential details of the model at the first and second levels (e.g. fixed, random or mixed effects; drift or auto-correlation).*
Effect(s) tested	*Define precise effect in terms of the task or stimulus conditions instead of psychological concepts and indicate whether ANOVA or factorial designs were used.*

Specify type of analysis: ☐ Whole brain ☐ ROI-based ☐ Both

Statistic type for inference (See Eklund et al. 2016)	*Specify voxel-wise or cluster-wise and report all relevant parameters for cluster-wise methods.*
Correction	*Describe the type of correction and how it is obtained for multiple comparisons (e.g. FWE, FDR, permutation or Monte Carlo).*

Models & analysis

n/a	Involved in the study
☐	☐ Functional and/or effective connectivity
☐	☐ Graph analysis
☐	☐ Multivariate modeling or predictive analysis

Functional and/or effective connectivity	*Report the measures of dependence used and the model details (e.g. Pearson correlation, partial correlation, mutual information).*
Graph analysis	*Report the dependent variable and connectivity measure, specifying weighted graph or binarized graph, subject- or group-level, and the global and/or node summaries used (e.g. clustering coefficient, efficiency, etc.).*
Multivariate modeling and predictive analysis	*Specify independent variables, features extraction and dimension reduction, model, training and evaluation metrics.*
